# Loss of P2X7 Receptor Plasma Membrane Expression and Function in Pathogenic B220^+^ Double-Negative T Lymphocytes of Autoimmune MRL/*lpr* Mice

**DOI:** 10.1371/journal.pone.0052161

**Published:** 2012-12-21

**Authors:** Sylvain M. Le Gall, Julie Legrand, Mohcine Benbijja, Hanaa Safya, Karim Benihoud, Jean M. Kanellopoulos, Pierre Bobé

**Affiliations:** 1 Institut Jacques Monod, Centre National de la Recherche Scientifique, Université Paris Diderot, Paris, France; 2 Institut André Lwoff, Centre National de la Recherche Scientifique, Université Paris-Sud, Villejuif, France; 3 Institut Gustave Roussy, Centre National de la Recherche Scientifique, Université Paris-Sud, UMR 8203, Villejuif, France; 4 Institut de Biochimie et Biophysique Moléculaire et Cellulaire, Centre National de la Recherche Scientifique, Université Paris-Sud, UMR 8619, Orsay, France; Beth Israel Deaconess Medical Center, United States of America

## Abstract

Lupus is a chronic inflammatory autoimmune disease influenced by multiple genetic loci including *Fas Ligand* (FasL) and *P2X7 receptor* (P2X7R). The Fas/Fas Ligand apoptotic pathway is critical for immune homeostasis and peripheral tolerance. Normal effector T lymphocytes up-regulate the transmembrane tyrosine phosphatase B220 before undergoing apoptosis. Fas-deficient MRL/*lpr* mice (*lpr* mutation) exhibit lupus and lymphoproliferative syndromes due to the massive accumulation of B220^+^ CD4^–^CD8^–^ (DN) T lymphocytes. The precise ontogeny of B220^+^ DN T cells is unknown. B220^+^ DN T lymphocytes could be derived from effector CD4^+^ and CD8^+^ T lymphocytes, which have not undergone activation-induced cell death due to inactivation of Fas, or from a special cell lineage. P2X7R is an extracellular ATP-gated cell membrane receptor involved in the release of proinflammatory cytokines and TNFR1/Fas-independent cell death. P2X7R also regulate early signaling events involved in T-cell activation. We show herein that MRL/*lpr* mice carry a *P2X7R* allele, which confers a high sensitivity to ATP. However, during aging, the MRL/*lpr* T-cell population exhibits a drastically reduced sensitivity to ATP- or NAD-mediated stimulation of P2X7R, which parallels the increase in B220^+^ DN T-cell numbers in lymphoid organs. Importantly, we found that this B220^+^ DN T-cell subpopulation has a defect in P2X7R-mediated responses. The few B220^+^ T cells observed in normal MRL^+/+^ and C57BL/6 mice are also resistant to ATP or NAD treatment. Unexpectedly, while P2X7R mRNA and proteins are present inside of B220^+^ T cells, P2X7R are undetectable on the plasma membrane of these T cells. Our results prompt the conclusion that cell surface expression of B220 strongly correlates with the negative regulation of the P2X7R pathway in T cells.

## Introduction

MRL/*lpr* mice develop a severe autoimmune syndrome that closely resembles human systemic lupus erythematosus (SLE), and exhibit a lymphoproliferative syndrome due to the *lpr* mutation of the death receptor Fas. The *lpr* mutation leads to the accumulation of a subset of TCRαβ^+^ B220^+^ CD4^–^CD8^–^ T lymphocytes (hereafter referred to as B220^+^ double negative (DN) T cells). The precise ontogeny of B220^+^ DN T cells is unknown. They could be derived either from CD4^+^ or CD8^+^ T cells, which despite antigen stimulation have not undergone activation-induced cell death phenomenon due to the defective *fas* gene [1], [2], or from a special cell lineage [3]. Accumulation of B220^+^ DN T cells in the *lpr* mouse appears to require the transcription factor eomesodermin [4]. Normal CD4^+^ or CD8^+^ effector T cells up-regulate the B220 isoform of CD45 (or CD45RABC) prior to apoptosis [5], [6]. CD45 is a transmembrane protein tyrosine phosphatase expressed at high levels on all nucleated hematopoietic cells [7]. When Fas or Fas ligand (FasL) expression is defective, effector T cells down-regulate CD4 or CD8 molecules but retain the expression of B220. Furthermore, *lpr* mutation results in a massive up-regulation of FasL on both T cells [8] and B cells [9], enabling them to kill Fas^+^ targets *in vitro*. The *lpr* mutation being leaky [10], FasL overexpression on MRL/*lpr* lymphocytes could be responsible for a non-antigen-specific autoimmune attack on organs expressing low levels of Fas. Indeed, we have demonstrated previously the ability of MRL/*lpr* T cells overexpressing FasL to induce non-antigen-specific killing *in vivo* [11], [12], and we have also shown that *in vivo* elimination of these FasL^+^ T cells using arsenic trioxide (As_2_O_3_) administration cured or prevented autoimmune diseases [13]. However, the *lpr* mutation cannot account for the entire autoimmune syndrome of MRL/*lpr* mice because C57BL/6 (B6) mice bearing the *lpr* mutation develop a lymphoproliferation, but a limited lupus-like syndrome [14]. Lupus is a polygenic disorder and a large number of susceptibility genes are identified in SLE and mouse models of spontaneous lupus [15], [16]. Interestingly, the purinergic receptor P2X7 (P2X7R) might be a susceptibility locus for lupus [17], [18].

P2X7R belongs to the P2X receptor family of ATP-gated cation channels [19]. Brief activation of P2X7R with high concentrations of ATP (>100 µM) opens cation-specific ion channels on the cell surface. Prolonged activation of P2X7R results in the formation of nonselective membrane pores, permeable to molecules of molecular mass up to 900 Da. Continuous ATP stimulation of P2X7R leads to cell death [20]–[22]. Depending on the cell type, numerous physiological functions have been attributed to P2X7R: maturation and secretion of inflammatory cytokines such as IL-1β, IL-18, IL-6 and TNF-α [23]–[26], shedding of the transmembrane molecules CD23 and L-selectin (CD62L) from lymphocytes [27] by activation of metalloproteinases ADAM10 and 17 [28], killing of different intracellular pathogens in macrophages [29], [30], and cell death [20]–[22]. P2X7R also regulates the early signaling events involved in T-cell activation. Upon antigen stimulation, T lymphocytes release intracellular ATP, which induces Ca^2+^ influx, NF-AT activation, and IL-2 production trough P2X7R activation [31]–[33].

Here, we report that the T-lymphocyte population from autoimmune MRL/*lpr* mice becomes more resistant with age to ATP-induced shedding of CD62L, pore formation, phosphatidylserine (PS) exposure, and cell death. This resistance is independent of the *P2X7R* gene polymorphism because the *P2X7R* allele in MRL/*lpr* and MRL^+/+^ mice is identical to the one from BALB/c mice, known to confer high sensitivity to stimulation by ATP [21]. The severe decrease in P2X7R activity in T lymphocytes parallels the increase in B220^+^ DN T lymphocyte numbers. The *in vivo* elimination of pathogenic B220^+^ DN T lymphocytes in MRL/*lpr* mice after treatment with As_2_O_3_ unmasked the activation of P2X7R by ATP in the remaining B220^–^ T-lymphocyte populations. Using a rabbit polyclonal antiserum against P2X7R, we were able to show that the resistance of B220^+^ T lymphocytes to ATP treatment is due to a dramatic decrease in P2X7R cell surface expression. Importantly, the few activated CD4^+^ and CD8^+^ T lymphocytes from normal MRL^+/+^ and B6 mice, which have upregulated B220 prior to apoptosis, are also resistant to ATP treatment, suggesting that B220 is a negative regulator of the ATP/P2X7R pathway in T cells. Our present data lead us to hypothesize that the defect in the homeostasis of activated T cells due to the inactivation of the Fas/FasL pathway is greatly amplified by the deregulated activity of P2X7R in the B220-expressing activated T cells. Since dysregulation of T cell homeostasis is emerging as a major contributor to autoimmune disease, we discuss the role of Fas, P2X7R and CD45 in the pathology of the autoimmune MRL/*lpr* strain.

## Materials and Methods

### Mice

Wild-type MRL/MpJ (MRL*^+/+^*), Fas deficient MRL/MpJ-*Fas^lpr/lpr^*/J (MRL/*lpr*), C57BL/6J (B6), Fas-deficient B6.MRL-*Fas^lpr/lpr^*/J (B6/*lpr*) and P2X7R-deficient C57BL/6 (B6*^P2X7R−/−^,* seventh backcross on B6) mice originally from The Jackson Laboratory (Bar Harbor, ME) were maintained at the SEAT (Service des Animaux Transgéniques) animal facility. All the experiments were conducted in accordance with French (décret n°87–848) and European Economic Community (directive 86/609/CEE) guidelines for the care of laboratory animals and approved by the Ethical Committee of the SEAT - UPS44 CNRS (Villejuif, France).

### Reagents

ATP (adenosine-5′-triphosphate), NAD (nicotinamide adenine dinucleotide), ATP/ADPase apyrase, PMA (phorbol myristate acetate), KN-62 (1-[N,O-bis(5-Isoquinolinesulfonyl)-N-methyl-L-tyrosyl]-4-phenylpiperazine), Arsenic trioxide (As_2_O_3_) were purchased from Sigma-Aldrich (St. Louis, MO). Ionomycin was from Calbiochem (EMD Biosciences Inc, San Diego, CA). Metalloprotease inhibitor GM6001 was purchased from Chemicon International (Temecula, CA), and inhibitor of zinc-dependent metalloproteinases Ro 31–9790 was kindly provided by Roche Palo Alto LLC (Palo Alto, CA).

### Genetic Immunizations of Rabbits with Plasmid DNA Encoding P2X7R

Rabbits were immunized against P2X7R by intramuscular electrotransfer of 400 µg (1 µg/µl in 0.9% NaCl) of the eukaryotic expression vector pcDNA3.1 containing mouse P2X7R cDNA. Animals were immunized three times at 3-week interval. Anti-P2X7R antisera were absorbed with spleen and thymus cells from B6*^P2X7R−/−^* mice. The specificity of these antisera was established by comparing immunostaining for mouse P2X7R on Human Embryonic Kidney (HEK) cells and HEK cells transfected with cDNA coding for mouse P2X7R and on T lymphocytes from P2X7R^+/+^ and P2X7R^−/−^ mice. While these rabbit anti-P2X7R antisera immunoprecipitate P2X7R from P2X7R^+/+^ cell lysates, they do not work in western-blot [34]. Thus, the rabbit polyclonal anti-P2X7R antisera recognize P2X7R in its conformational form only, and are used for flow cytometric analysis.

### Cell Preparation and Cell-surface Phenotyping

Spleen and lymph node (LN) cell suspensions prepared from 2 to 5-mo-old mice were subjected to Ficoll-Hypaque density gradient centrifugation to remove erythrocytes and dead cells. For phenotyping by flow cytometry and cell purification by Fluorescence Activated Cell Sorting (FACS), spleen and LN cells were stained using either FITC-, PE- or biotin-conjugated mAb: rat anti-CD90.2, anti-CD4, anti-CD8, anti-B220, anti-CD19, anti-CD39 (PharMingen, BD Biosciences or eBioscience, San Diego, CA). Plasma membrane-associated ADP-ribosyltransferase (ART)2.2 enzyme was detected using FITC-conjugated rat anti-mouse ART2.2 mAb (PharMingen). P2X7R was detected using rabbit polyclonal anti-P2X7R sera, generated by genetic immunization, and biotin-conjugated goat F(ab’)_2_ anti-rabbit IgG absorbed against mouse immunoglobulins (SouthernBiotech, Birmingham, AL). Rat IgG2a mAb was used as the isotype control (PharMingen). Use of mAb to mouse Fcγ receptor (PharMingen) avoided non-specific antibody binding.

### Assays for CD62L Shedding, Pore Formation, PS Exposure and Calcium Influx

Spleen or LN cell suspensions were treated with ATP for 30 to 45 min at 37°C. In some experiments lymphoid cells were pretreated with P2X7R antagonist KN-62 for 15 min at 37°C before stimulation with ATP, NAD or PMA plus Ionomycin (PMA/Iono). Cells were then washed in PBS, and stained for 30 min on ice with phenotype-specific fluorescent mAbs, and with either FITC-conjugated anti-CD62L mAb (PharMingen), YO-PRO-1 fluorescent probe (Invitrogen-Molecular Probes, Cergy Pontoise, France), or FITC-conjugated Annexin V (Invitrogen-Molecular Probes) to assess CD62L shedding, pore formation, or cell surface PS exposure, respectively, by flow cytometry. For the measurement of calcium influx by flow cytometry, cells were loaded for 30 min at 37°C with calcium-reactive fluorescence probe, Oregon Green 488 BAPTA-1 (Invitrogen-Molecular Probes). Cells were stained with phenotype-specific fluorescent mAbs (30 min on ice) prior to stimulation with ATP.

### Cell Death Analysis

Spleen cell suspensions were treated with ATP or NAD at 37°C for 4 h. Cells were then stained with phenotype-specific fluorescent mAbs, and with 7-aminoactinomycin D (7-AAD, PharMingen) to assess cell viability by flow cytometry.

### P2X7R Sequencing

Total RNA was extracted with RNeasy Kit (Qiagen, Courtaboeuf, France) from individual MRL^+/+^ and MRL/*lpr* spleens, and 1 µg of each sample was reverse-transcribed into cDNA using a cDNA-synthesis kit (New England Biolabs, Evry, France). PCR were performed on cDNA using the FastStart High Fidelity PCR System (Roche Diagnostics, Mannheim, Germany) and P2X7R forward primer (5′-ATG CCG GCT TGC TGC AGC TGG AAC GAT GTC TT-3′) and P2X7R reverse primer (5′-TCA GTA GGG ATA CTT GAA GCC ACT GTA CTG CCC-3′). PCR products were gel-purified using the QIAquick Gel Extraction Kit (Qiagen), and nucleotide sequences were determined by MWG Biotech Company (Ebersberg, Germany).

### Real-time RT-PCR

RNAs were extracted with RNeasy kit (Qiagen) from whole LN cells, CD90^–^CD19^+^ B cells as well as B220^+^ and B220^–^ CD19^–^CD90^+^ T-cell subsets purified from LNs of individual B6, MRL^+/+^ and MRL/*lpr* mice by FACS. One µg of each sample was reverse-transcribed into cDNA using a cDNA-synthesis kit (New England Biolabs). PCR was conducted with the LightCycler system using the DNA-binding dye SYBR Green for the detection of PCR products (Roche Diagnostics). DNA amplification was performed using P2X7R forward primer (5′-AGC ACG AAT TAT GGC ACC GT-3′) and P2X7R reverse primer (5′-CCC CAC CCT CTG TGA CAT TCT-3′). After being heated at 95°C for 8 min, cDNA were amplified for 40 cycles, each cycle consisting of 95°C for 15 s, 60°C for 5 s, and 72°C for 8 s. For melting curve analysis, samples were heated to 95°C at a transition rate of 0.1°C/s with continuous fluorescence readings. The specificity of PCR products was confirmed by melting curve analysis and by running samples on agarose gels. Specific cDNA was quantified by standard curves based on known amounts of PCR-amplified P2X7R cDNA, which were 10 pg, 2 pg, 0.40 pg, 0.080 pg, 0.016 pg, 0.0032 pg and 0.00064 pg. The standards and the samples were simultaneously amplified using the same reaction master mixture.

### Western Blot Analysis

LN cells from B6*^P2X7R−/−^*, MRL*^+/+^*, MRL/*lpr* mice as well as FACS-purified B220^+^ and B220^–^ T-cell subsets from individual MRL/*lpr* LNs were lysed in Tris-buffered saline (TBS) (50 mM Tris pH 7.5, 150 mM NaCl) containing 1% NP-40, 1% sodium deoxycholate, 0.1% SDS and protease inhibitors (Roche Diagnostics) for 15 min on ice. Cell lysates were centrifuged at 12,000 *g* for 15 min at 4°C, and protein concentration in the supernatants was determined using the BCA protein assay kit (Pierce, Rockford, IL). Equal amounts of proteins from each lysate were separated on NuPAGE 4–12% Bis-Tris gradient gels (Invitrogen) under nonreducing condition, and then electrotransferred to nitrocellulose membranes (Whatman/Schleicher & Schuell, Sigma-Aldrich). The membranes were blocked in TBS containing 3% nonfat dry milk and 0.2% Tween 20 for 1 h at room temperature and subsequently incubated overnight at 4°C with affinity purified rabbit anti-P2X7R antibodies recognizing residues 576 to 595 of P2X7R (1∶1000; Alomone Laboratories, Jerusalem, Israel). Finally, blots were incubated with HRP-conjugated goat anti-rabbit IgG (1∶2500; Pierce) for 1 h at room temperature, and protein bands were visualized by enhanced chemiluminescence reagents (SuperSignal West Dura Extended Duration Substrate, Pierce). Washings were performed in TBS containing 0.05% Tween 20. For reprobing with rabbit anti-actin antibodies (1∶500; Sigma-Aldrich), the blots were first incubated in stripping buffer (Pierce) for 10 min at room temperature.

### Statistical Analysis

Data are reported as mean ± SE. Comparisons between untreated and ATP- or NAD-treated groups were made by Student’s t-test. Statistical difference was accepted at *P*≤0.05.

## Results

### Impaired P2X7R Activity in T Cells from Autoimmune MRL/lpr Mice

**Figure 1:**
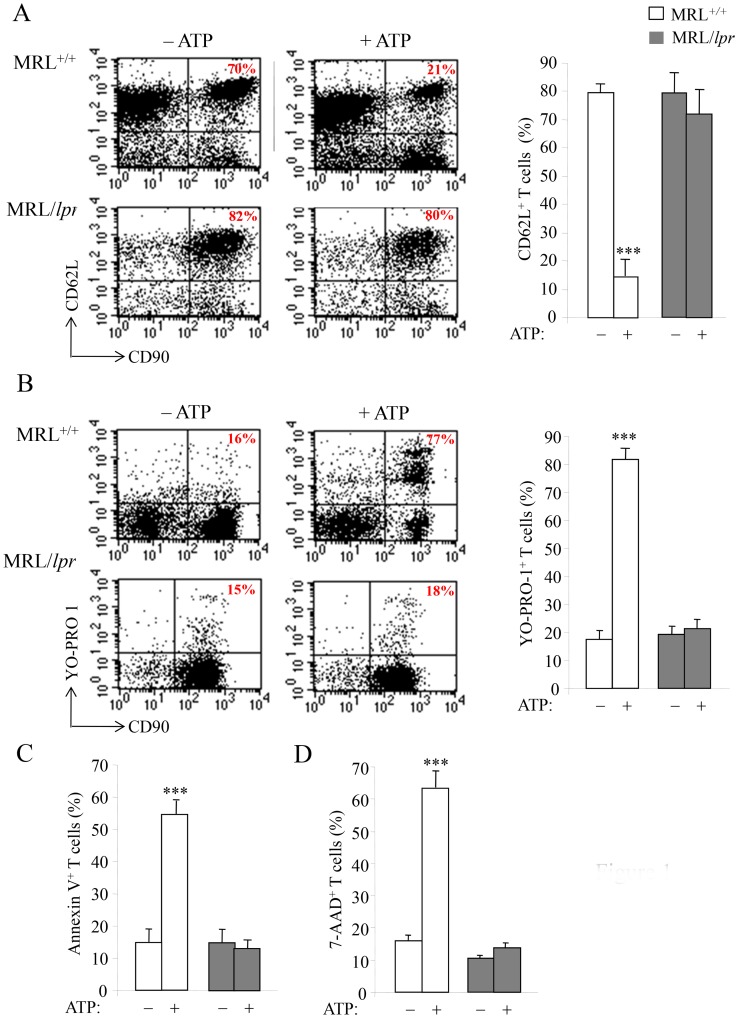
P2X7R activity in T lymphocytes from normal MRL^+/+^ and autoimmune MRL/lpr mice. Spleen cells from 3 to 4-mo-old MRL*^+/+^* (□) and MRL/*lpr* (▪) mice (n = 5 mice/group) were incubated with or without 500 µM ATP for 45 min (A–C) or 4 h (D) at 37°C. Cells were then double-stained with anti-CD90 mAb and either anti-CD62L mAb, YO-PRO-1, Annexin V or 7-AAD to assess in T cells by flow cytometry: (A) CD62L shedding; (B) pore formation; (C) cell surface PS exposure; (D) cell death. Numbers reported in the dot plots indicate the percentages of CD62L^+^CD90^+^ or YO-PRO-1^+^CD90^+^ cells in the gated CD90^+^ T-cell population. Histograms correspond to the mean percentages ± SE (n = 5 mice/group) of double-stained cells in the gated CD90^+^ T-cell population. Asterisks denote statistically significant differences (***p≤0.001) between ATP-stimulated and unstimulated groups.

**Figure 2:**
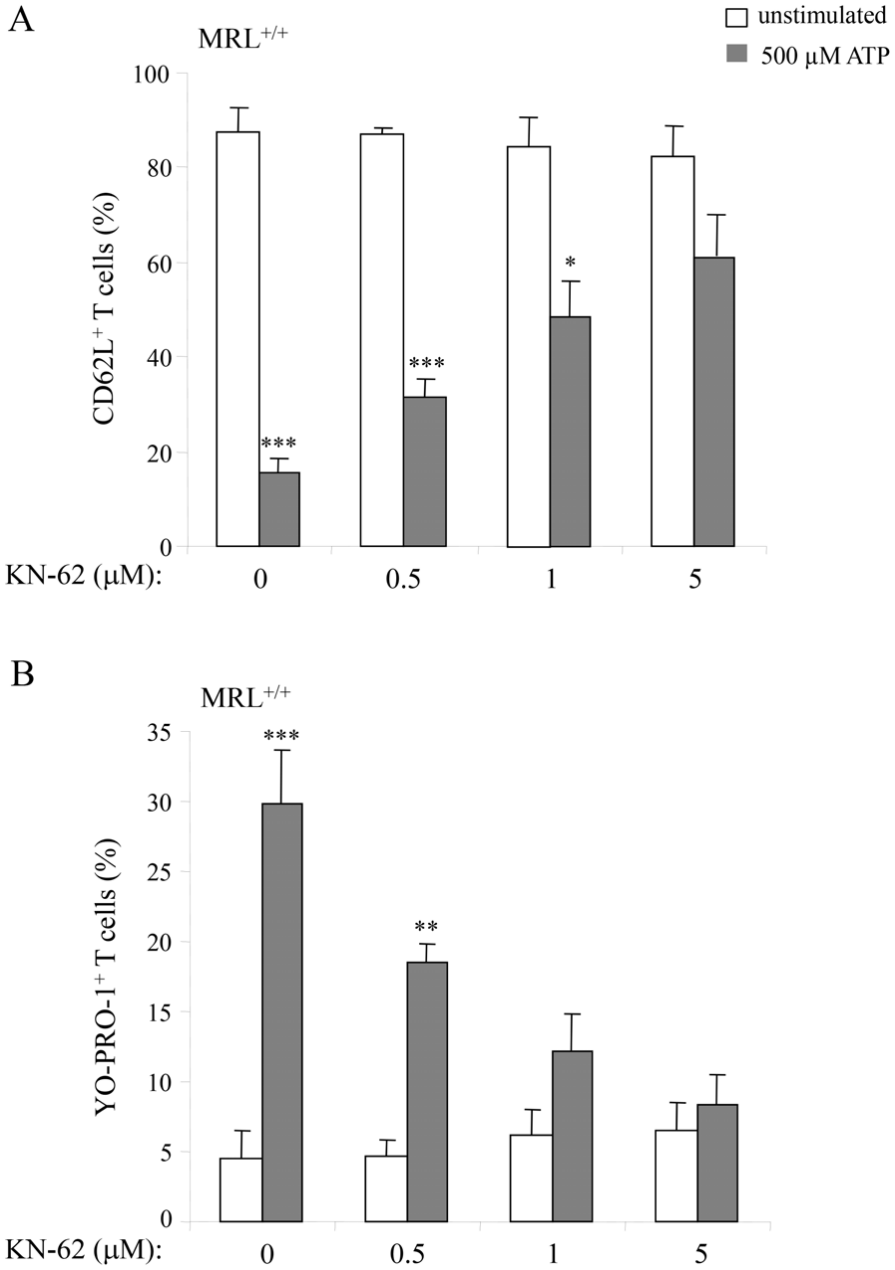
P2X7R antagonist KN-62 inhibits ATP-induced CD62L shedding and pore formation in MRL^+/+^ T lymphocytes. Spleen cells from 3 to 4-mo-old MRL*^+/+^* mice (n = 4 mice/group) were preincubated for 15 min at 37°C with or without 0.5, 1 and 5 µM KN-62 and then stimulated with 500 µM ATP for 30 min. Cells were then double-stained with anti-CD90 mAb and either anti-CD62L or YO-PRO-1 to assess by flow cytometry: (A) CD62L shedding or (B) pore formation in the gated CD90^+^ T-cell population stimulated (▪) or not (□) with ATP. Asterisks denote statistically significant differences between ATP-stimulated and unstimulated groups: *p≤0.05; **p≤0.01; ***p≤0.001.

P2X7R stimulation by ATP results in PS exposure, pore formation, shedding of transmembrane molecules and cell death. Here we compared the sensitivity to ATP stimulation of T cells from autoimmune MRL/*lpr* mice with their counterparts from normal B6 and MRL^+/+^ mice. Measurements of pore formation (YO-PRO-1 uptake), CD62L shedding, PS exposure on cell surface (Annexin V staining) and cell death (7-AAD staining) in ATP-treated spleen cells were performed simultaneously with cell phenotyping. Stimulation by 500 µM ATP of spleen cells from B6 (not shown) and MRL^+/+^ mice triggered CD62L shedding (Fig. 1A), YO-PRO-1 uptake (Fig. 1B), and Annexin V (Fig. 1C) and 7-AAD (Fig. 1D) staining in CD90^+^ T cells. In contrast, the CD90^+^ T cells from autoimmune MRL/*lpr* did not respond to 500 µM ATP (Figs. 1A, 1B, 1C and 1D). Furthermore, using the ATP/ADP-degrading enzyme apyrase and KN-62, a potent pharmacological antagonist of P2X7R, we confirmed that P2X7R stimulation is implicated in CD62L shedding and pore opening in MRL^+/+^ T cells. Upon treatment with 500 µM ATP, 79% of MRL^+/+^ T cells released CD62L and 45% incorporated YO-PRO-1 *vs*. 12% and 0.45%, respectively, when cells were treated with 500 µM ATP plus 20 U/ml apyrase. Similarly, pre-treatment of MRL^+/+^ spleen cells by KN-62 inhibited ATP-induced CD62L shedding (Fig. 2A) and YO-PRO-1 uptake (Fig. 2B) in T cells in a dose-dependent manner.

**Figure 3:**
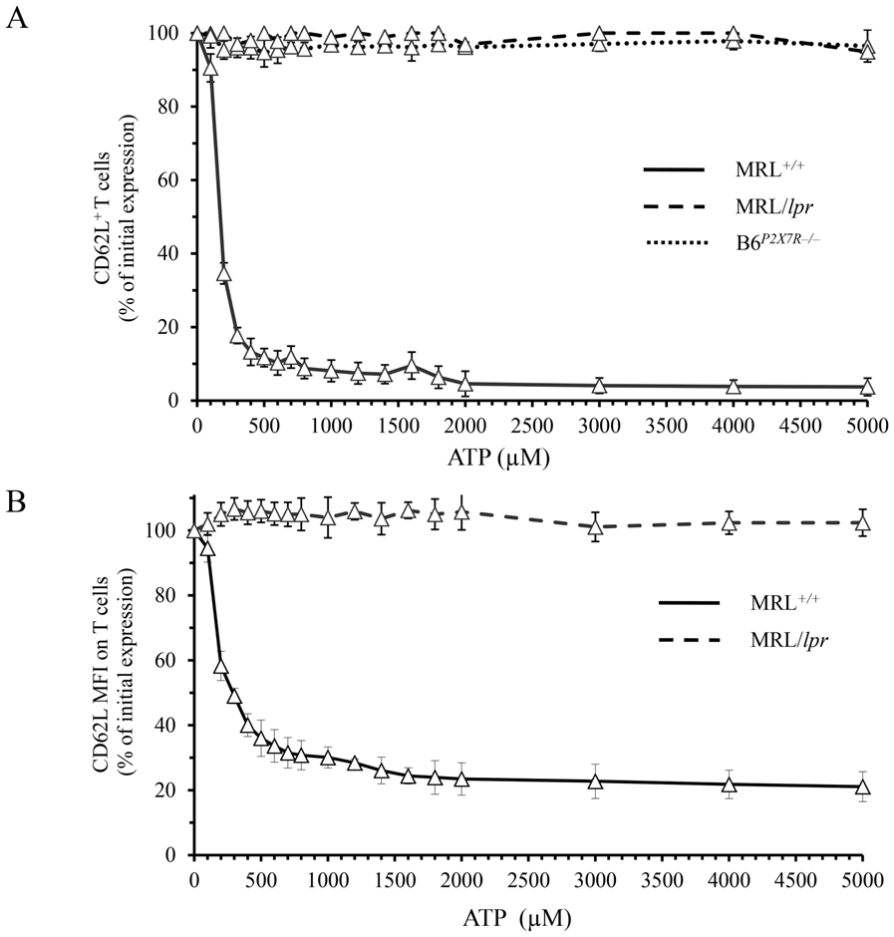
Dose–response experiments for ATP-induced CD62L shedding on T lymphocytes. Spleen cells from 3 to 4-mo-old MRL*^+/+^* (*solid line*), MRL/*lpr* (*dashed line*) and P2X7-deficient B6*^P2X7R−/−^* (*dotted line*) mice (n = 3 mice/group) were treated for 45 min at 37°C with doses of ATP ranging from 100 to 5000 µM. Spleen cells were then triple-stained with anti-CD90, anti-CD19 and anti-CD62L mAb to assess by flow cytometry: (A) the percentage of CD62L-expressing CD19^–^CD90^+^ T cells and (B) MFI of CD62L on CD19^–^CD90^+^ T cells. Results are expressed as the mean percentage of initial expression ± SE.

MRL^+/+^ spleen cells required for stimulation a high concentration of ATP because P2X7R displays a low affinity to this ligand (typically ≥100 µM) compared with other P2XR [35]. To determine whether T cells from MRL/*lpr* mice require an even higher dose of ATP to be stimulated than those from MRL^+/+^, we conducted dose–response experiments with ATP ranging from 100–5000 µM (Fig. 3A). In MRL^+/+^ T cells, CD62L shedding was observed at a half-maximal effective concentration (EC_50_) of 170 µM ATP, while MRL/*lpr* T cells were totally resistant to stimulation by ATP, at any dose tested. Consistently, in B6*^P2X7R−/−^* T cells, ATP had no effect on CD62L shedding, indicating that this effect is dependent on P2X7R (Fig. 3A). The resistance of MRL/*lpr* T cells to ATP-induced CD62L shedding is also obvious when CD62L expression is depicted as an average fluorescence intensity per cell (mean fluorescence intensity, MFI) (Fig. 3B) instead of the percentage of CD62L^+^ T cells (Figs. 1, 2 and 3A). Indeed, even at the highest dose of ATP, CD62L MFI values in MRL/*lpr* T lymphocytes remained unaffected by the treatment with ATP (Fig. 3B).

NAD also acts as a ligand for P2X7R through ART2.2 enzyme, which catalyzes the transfer of ADP-ribose from NAD to various cell surface proteins, among them the P2X7R [36]. Stimulation by 50 µM NAD of MRL^+/+^ and MRL*/lpr* spleen cells induced CD62L shedding and PS exposure only on MRL^+/+^ T cells (Fig. S1). As found with ATP, NAD is unable to promote P2X7R pathway activation in MRL/*lpr* T cells. This absence of CD62L shedding and PS exposure in NAD-treated MRL/*lpr* T cells is not due to a lower expression of ART2.2 because the percentage of ART2.2^+^-expressing T cells was similar in MRL*/lpr* (33±1.7%) and MRL^+/+^ (29±2.5%) spleen cells as analyzed by flow cytometry using anti-ART2.2 mAb.

Taken altogether, these data show that ATP- or NAD-stimulated P2X7R events are dramatically impaired in T cells from autoimmune MRL*lpr* mice.

### PMA/Iono Induces Shedding of CD62L in ATP-insensitive MRL/lpr T Cells

**Figure 4:**
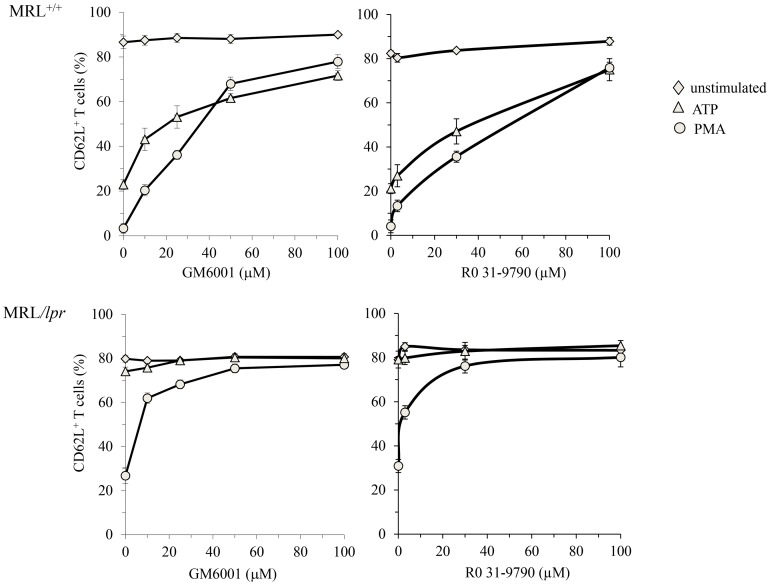
Metalloprotease inhibitors GM6001 and Ro 31–9790 inhibit PMA/Iono- and ATP-induced shedding of CD62L on MRL^+/+^ T lymphocytes. Spleen cells from MRL*^+/+^* and MRL/*lpr* mice (n = 5 mice/group) were preincubated with or without 10, 25, 50, 100 µM GM6001 or 3, 30, 100 µM Ro 31–9790 for 15 min at 37°C, and then either left unstimulated (□) or stimulated with 500 µM ATP (▴) or 5 ng/ml PMA plus 0.5 µg/ml Ionomycin (○) for 45 min at 37°C. Cells were subsequently stained with anti-CD90 and anti-CD62L mAb. Cell surface expression of CD62L was assessed in the gated CD90^+^ T cells by flow cytometry.

Treatment of leukocytes with ATP or PMA is known to induce CD62L shedding via activation of the zinc-dependent membrane metalloprotease ADAM17 or TACE (TNF-α converting enzyme) [27], [28]. However, PMA acts independently of the P2X7R pathway. Unlike ATP treatment, which activates CD62L shedding in T cells from MRL^+/+^, but not from MRL/*lpr* mice, PMA/Iono treatment induces CD62L shedding in T cells from both MRL^+/+^ and MRL/*lpr* mice. The shedding of CD62L by ATP in MRL^+/+^ T cells, or by PMA/Iono in MRL^+/+^ and MRL/*lpr* T cells was prevented by the metalloprotease inhibitors GM6001 and Ro 31–9790 (Fig. 4), in a similar dose dependent-manner. These data show that while ADAM17 is present in T cells from both MRL^+/+^ and MRL/*lpr* mice, its activation by P2X7R signaling [28] is dramatically impaired in MRL/*lpr* T cells, and also partially reduced upon PMA/Iono treatment. This reduced processing activity of CD62L in MRL/*lpr* T cells could be due to a general defect or repression of signalling activities in this strain.

### The MRL Genetic Background Carries a High Sensitivity Allele of P2X7R

P2X7R is highly polymorphic [26]. In the common laboratory mouse strains B6 and DBA/2, and in the lupus-prone NZB mice, an allelic variation (P451L) in the C-terminal tail of P2X7R confers a low sensitivity to stimulation by ATP compared with BALB/c or NZW mice [17], [21]. We determined the *P2X7R* allele present in the MRL genetic background by sequencing RT-PCR-amplified P2X7R products obtained from spleen cells of 7 independent MRL^+/+^ and MRL/*lpr* mice (data not shown). We found that the more responsive allele of P2X7R, the P451 isoform [21], is carried by the MRL genetic background and present in both MRL*^+/+^* and MRL/*lpr* mice. Thus, an allelic variation cannot explain the differences in the levels of P2X7R signaling found between MRL*^+/+^* and MRL/*lpr* mice.

### ATP-induced P2X7R Activation is Blocked in B220^+^ T Cells Only

**Figure 5:**
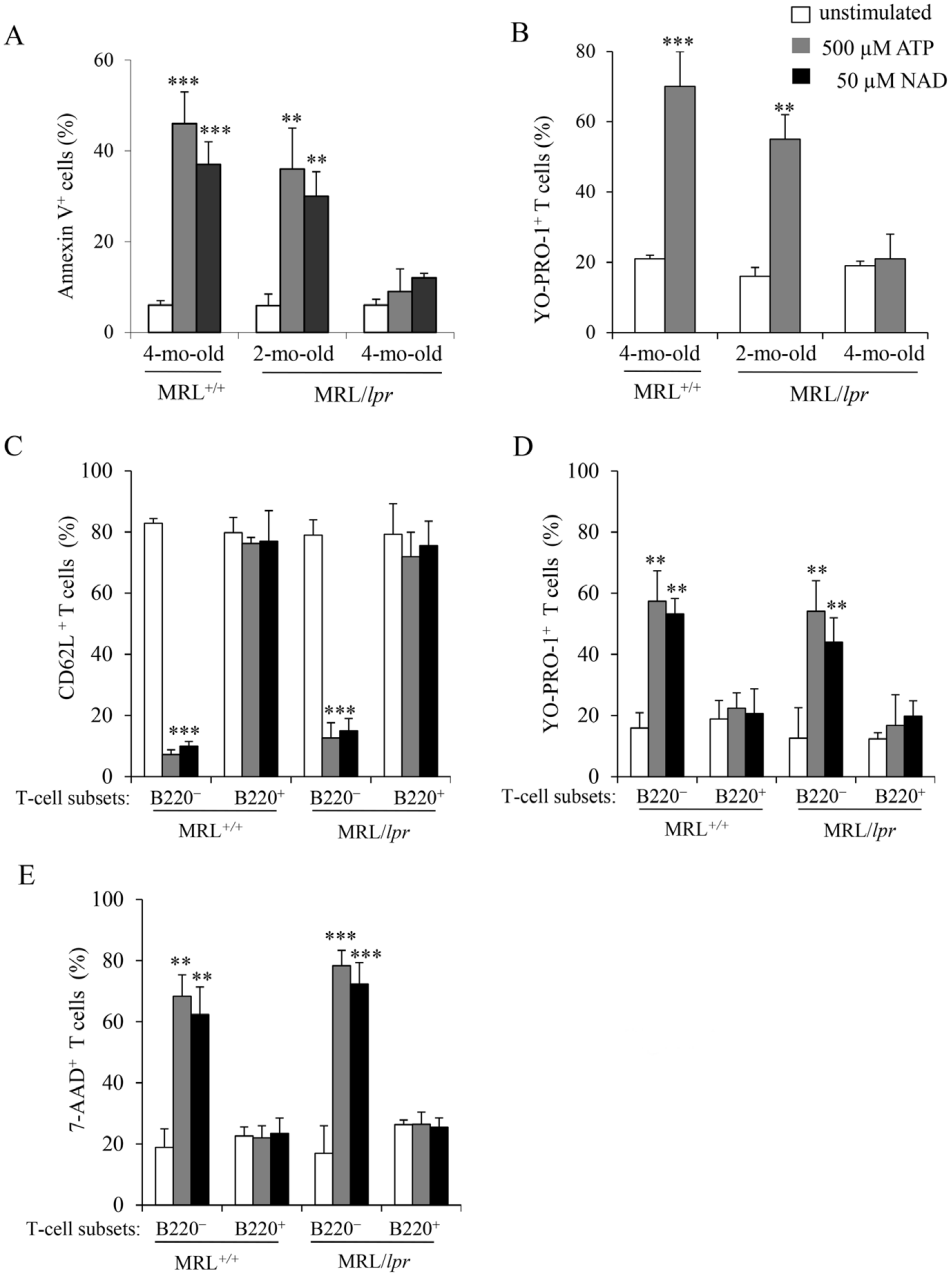
P2X7R activity in T lymphocytes from MRL/lpr mice at early and late stages of the disease, and in B220^+^ and B220^–^ T lymphocytes from MRL^+/+^ and MRL/lpr mice. (A and B) Spleen cells from 4-mo-old MRL*^+/+^* and 2- and 4-mo-old MRL/*lpr* mice (n = 3 mice/group) were either left unstimulated (□) or stimulated with 500 µM ATP (▪) or 50 µM NAD (▪) for 45 min at 37°C. Cells were then double-stained with anti-CD90 mAb and either Annexin V or YO-PRO-1 to assess PS exposure and pore formation, respectively, in the gated CD90^+^ T-cell population by flow cytometry. (C–E) Spleen cells from 3 to 5-mo-old MRL^+/+^ and MRL*/lpr* (n = 5 mice/group) were either left unstimulated (□) or stimulated with 500 µM ATP (▪) or 50 µM NAD (▪) for 45 min at 37°C. Cells were then triple-stained with anti-CD90, anti-B220 and either anti-CD62L mAb, YO-PRO-1 or 7-AAD to assess CD62L shedding (C), pore formation (D) or cell death (E) in B220^–^CD90^+^ and B220^+^CD90^+^ T-cell subsets. Asterisks denote statistically significant differences between ATP- or NAD-stimulated and unstimulated groups: **p≤0.01; ***p≤0.001.

Unexpectedly, when comparing the responses of MRL/*lpr* and MRL^+/+^ spleen cells to ATP or NAD treatment measured by PS exposure (Fig. 5A) and pore formation (Fig. 5B), we found that the deficiency of P2X7R activity in MRL/*lpr* mice is age-related. Remarkably, the lack of ATP or NAD responsiveness in MRL/*lpr* mice can be reproducibly observed at later stages of the disease (4-mo-old), but is not seen in younger MRL/*lpr* mice (2-mo-old) at earlier stages of the autoimmune syndrome (Figs. 5A and 5B). Since MRL^+/+^ (4-mo-old) spleen T cells respond to ATP or NAD treatment, the weaker response of spleen T cells from 4-mo-old MRL/*lpr* mice to stimulation by ATP or NAD is probably due to intrinsic change in the lymphocyte subsets *i.e* the appearance of high percentage of B220^+^ DN T cells in the spleen of MRL/*lpr* mice. The B220^+^ DN T-cell subset is present at a very low frequency in spleen and LNs from 1-mo-old MRL/*lpr* mice, and the frequency of this subset increases with age to become predominant in 3 to 4-mo-old MRL*/lpr* mice (Table 1). Since As_2_O_3_ specifically eliminates B220^+^ DN T cells in MRL/*lpr* mice as we had shown previously [13], we examined whether T cells from As_2_O_3_-treated MRL/*lpr* mice remained refractory to P2X7R stimulation, or whether they behaved like MRL^+/+^ T cells when challenged with ATP. In experiments starting at 2 months of age, MRL^+/+^ and MRL/*lpr* mice were treated with a daily injection of As_2_O_3_ (5 µg/g body weight) or PBS for two months. As shown above, spleen cells from 4-mo-old PBS-treated MRL/*lpr* mice were resistant to 500 µM ATP. In contrast, spleen from 4-mo-old As_2_O_3_-treated MRL/*lpr* mice totally free of B220^+^ DN T cells responded to ATP treatment as efficiently as MRL^+/+^ T cells (Fig. S2), indicating that the lack of responsiveness to stimulation by ATP or NAD observed in T cells from MRL/*lpr* mice at later stages of the disease is due to the accumulation of B220^+^ DN T cells with impaired P2X7R functions.

**Table 1:** Percentages of the different T-cell subsets in LNs from MRL^+/+^, MRL/lpr, B6 and B6/lpr mice.

T-cell subsets	MRL^+/+^ (n = 15)*	“young” MRL/*lpr* (n = 10)**	“old” MRL/*lpr* (n = 15)*	B6 (n = 5)***	B6/*lpr* (n = 5)***
**B220^–^CD4^+^**	47.38±2.50	49.11±3	12.56±3.89	51.05±1.25	35.51±5.25
**B220^–^CD8^+^**	45.22±1.55	33.15±5.3	14.16±3.45	44.10±2.22	31.12±4.75
**B220^+^CD4^+^**	4.62±1.45	4.02±1.98	3.43±2.55	3.25±0.95	7.9±1.06
**B220^+^CD8^+^**	2.78±0.81	2.91±0.97	0.85±0.65	1.58±0.31	1.63±0.46
**B220^+^CD4^–^CD8^–^**	1.37±0.25	13.39±4.2	70.40±11.55	1.06±0.12	26.2±4.25

* 3 to 4-mo-old MRL^+/+^ and MRL/*lpr* mice, *n* represents the number of mice per group.

** 1½ to 2-mo-old MRL/*lpr* mice.

*** 5-mo- old B6 and B6/lpr mice.

**Figure 6:**
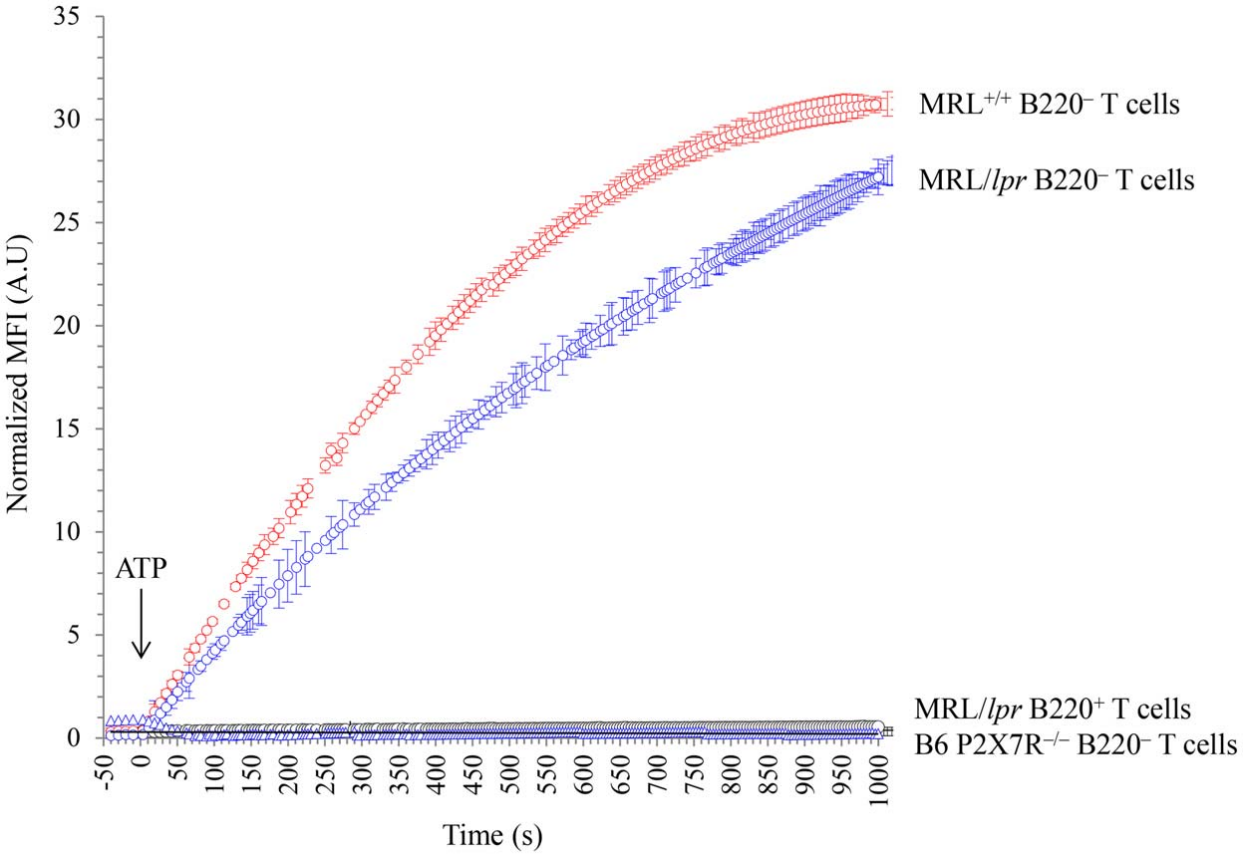
Real-time kinetic analysis of calcium influx in B220^–^ and B220^+^ T lymphocytes stimulated with ATP. Spleen cells from 4-mo-old MRL*^+/+^* (*red*) MRL/*lpr* (*blue*) and B6*^P2X7−/−^* (*black*) mice (n = 3 mice/group) were loaded with calcium-reactive fluorescence probe, Oregon Green 488 BAPTA-1. After loading, cells were stained with anti-CD90, anti-CD19 and anti-B220 mAbs. Baseline MFI of the calcium probe was recorded in the gated B220^–^CD90^+^ (○) and B220^+^CD90^+^ (▵) T cells during 50 s by flow cytometry, and was followed by the addition of 500 µM ATP (at time t0, arrow). Changes over time in MFI values of the calcium probe were monitored in the T-cell subsets by flow cytometry. MFI values were normalized by subtracting the baseline MFI.

**Figure 7:**
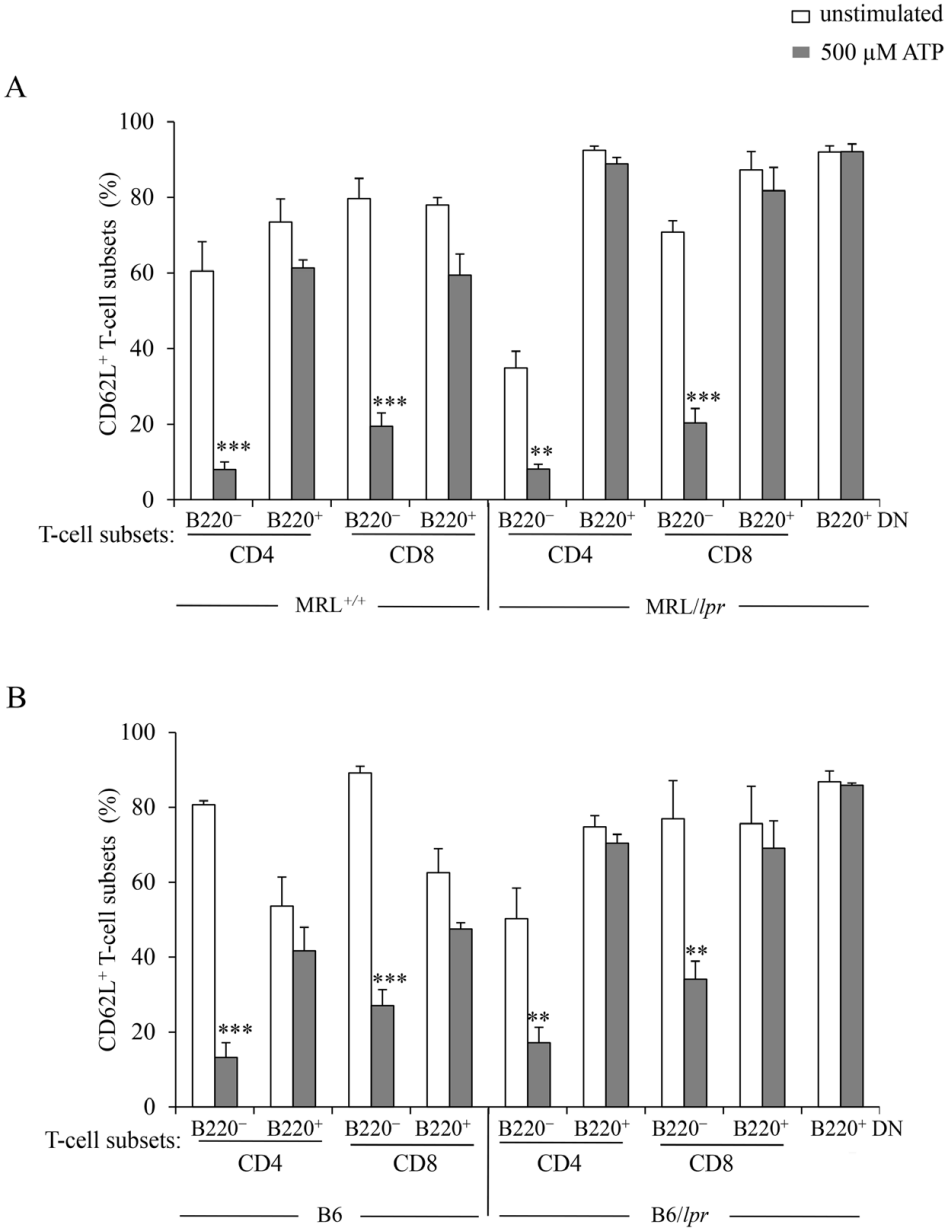
ATP-induced shedding of CD62L in CD4^+^, CD8^+^ and DN T lymphocytes expressing the transmembrane tyrosine phosphatase B220. Spleen cells from 3 to 5-mo-old MRL*^+/+^*, MRL*/lpr*, B6 and B6/*lpr* mice (n = 5 mice/group) were either left unstimulated (□) or stimulated with 500 µM ATP (▪) for 45 min at 37°C. Spleen cells were then stained with anti-CD90, anti-B220, anti-CD62L and anti-CD4 or anti-CD8 mAb to assess CD62L expression on B220*^–^* and B220^+^ CD90^+^ T cells (either CD4^+^ or CD8^+^) from MRL*^+/+^*, MRL*/lpr*, B6 and B6/*lpr* mice as well as on B220^+^ DN CD90^+^ T cells from MRL*/lpr* and B6/*lpr* mice. Asterisks denote statistically significant differences between ATP-stimulated and unstimulated groups: **p≤0.01; ***p≤0.001.

During aging B220^+^ DN T cells accumulate in *lpr* LNs and spleen and dilute out conventional T cells. Thus, B220^+^ DN T cells represent 70.40±11.55% and 26.2±4.25% of the T-cell population in 3 to 4-mo-old MRL/*lpr* mice and 5-mo-old B6*/lpr* mice, respectively. As expected, a small number of B220^+^ T cells (either CD4^+^, CD8^+^ or DN) were found in 4-mo-old wild-type MRL^+/+^ and B6 mice (Table 1). Their specific behaviour regarding stimulation by ATP is masked by the response of B220^–^ T cells. Therefore, we have examined the activity of P2X7R in B220^–^CD90^+^ and B220^+^CD90^+^ T-cell subsets from normal MRL*^+/+^* mice and autoimmune MRL*/lpr* mice. Stimulation by ATP or NAD of the B220^–^CD90^+^ T-cell subset from MRL/*lpr* and MRL*^+/+^* mice triggered CD62L shedding (Fig. 5C), pore formation (Fig. 5D), cell death (Fig. 5E), and calcium influx (Fig. 6). P2X7R is indeed involved in these cellular events because pre-treatment of MRL/*lpr* spleen cells by the P2X7R antagonist KN-62 inhibited ATP-induced CD62L shedding and pore formation in B220^–^CD90^+^ T cells in a dose-dependent manner (Fig. S3). In contrast, B220^+^CD90^+^ T cells are not activated by ATP or NAD in any mouse strain tested (MRL^+/+^, MRL/*lpr*, B6, B6/*lpr*) (Figs. 5C, 5D, 5E, 6 and 7). Furthermore, we compared the sensitivity to ATP of B220^–^CD4^+^ and B220^–^CD8^+^ T cells from MRL^+/+^, MRL/*lpr*, B6 and B6*/lpr* mice with their counterparts expressing the B220 molecule (Fig. 7A and 7B). Importantly, whatever the phenotype of the CD90^+^ T cells (either CD4^+^ or CD8^+^) and their genetic origin (either MRL^+/+^, MRL/*lpr*, B6 or B6*/lpr*), the cell surface expression of B220 molecules strictly correlated with an impaired cleavage of CD62L following stimulation by ATP, which indicates an absence of P2X7R signaling.

### CD39 Expression Levels on B220^+^ and B220^–^ T Cells

**Figure 8:**
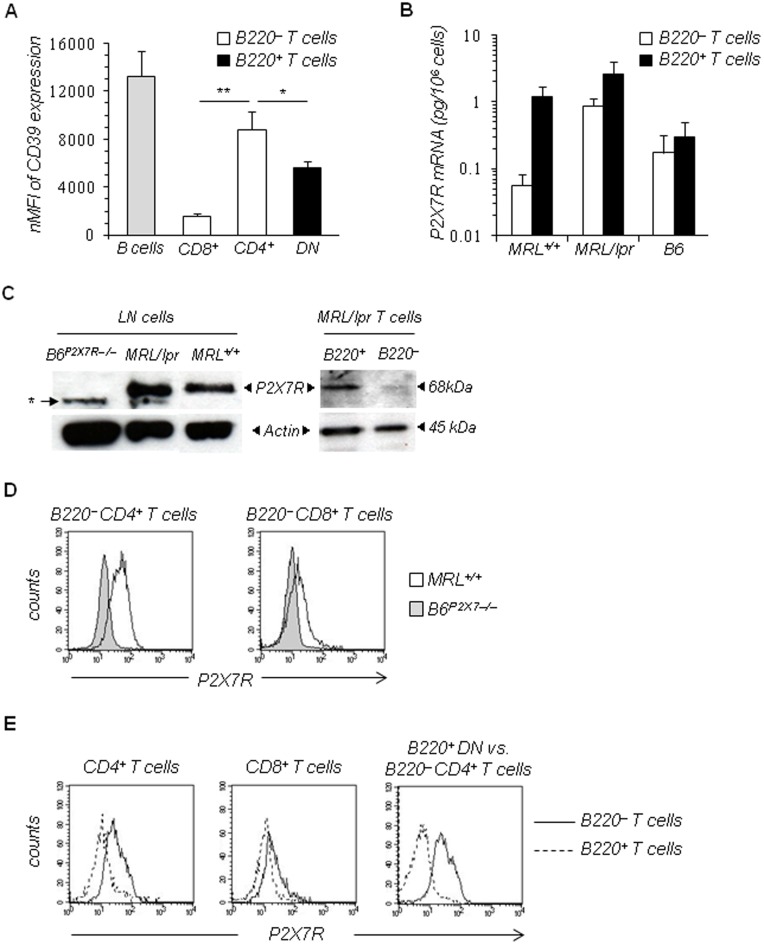
Expression of P2X7R and CD39 in T-cell subsets. (A) The levels of CD39 expression on B220^–^ (either CD4^+^ or CD8^+^) (□) and B220^+^ DN (▪) T-cell subsets as well as on CD19^+^ B cells (▪) from MRL/*lpr* mice (n = 3) were analyzed by flow cytometry. Results shown on histogram are normalized MFI (nMFI) calculated as MFI of positive cells × percentage of positive cells. Asterisks denote statistically significant differences between B220^+^ DN T cells or B220^–^CD8^+^ T cells and B220^–^CD4^+^ T cells: *p≤0.05; **p≤0.01. (B) P2X7R mRNA expression was analyzed in FACS-purified B220^–^ (□) and B220^+^ (▪) CD19^–^CD90^+^ T-cell subsets from individual B6, MRL^+/+^ and MRL/*lpr* mice by real-time RT-PCR. Specific P2X7R cDNA was quantified by standard curves based on known amounts of PCR-amplified P2X7R cDNA. Data are expressed as amount (pg) of P2X7R RNA per cell, and histograms represent the mean ± SE of three independent experiments (n = 8 mice/group). (C) P2X7R protein levels in whole LN cells from 3 to 4-mo-old MRL*^+/+^*, MRL/*lpr* and B6*^P2X7−/−^* mice, and FACS-sorted B220^–^ and B220^+^ CD19^–^CD90^+^ T-cell subsets from MRL/*lpr* mice were analyzed by western blotting using affinity-purified rabbit anti-P2X7R polyclonal antibodies (1∶1000; Alomone Laboratories). A non-specific band (*) is frequently observed with this polyclonal antibody [22]. The blot was stripped and reprobed with anti-actin mAb. (D and E) Flow cytometric analysis of P2X7R expression levels on B220^–^ and B220^+^ T-cell subpopulations. Spleen cells from MRL*^+/+^*, MRL/*lpr* and B6*^P2X7R−/−^* mice (n = 3 mice/group) were stained with anti-CD90 mAb, anti-B220 mAb, rabbit polyclonal anti-P2X7R antiserum (1∶100) generated by genetic immunization [34] and with either anti-CD4 mAb, anti-CD8 mAb or anti-CD4 plus anti-CD8 mAbs. (D) Overlay histograms showing the expression levels of P2X7R on gated CD4 T cells or CD8 T cells from MRL*^+/+^* mice (open histogram) and B6*^P2X7R−/−^* mice (shaded histogram). (E) Overlay histograms comparing the expression levels of P2X7R on B220^–^ (–) and B220^+^ (–) T cells, either CD4^+^ (*left*) or CD8^+^ (*middle*), from MRL/*lpr* mice. *Right*, overlay histogram comparing the expression levels of P2X7R on B220^+^ DN T cells (–) and B220^–^ CD4^+^ T cells (–) from MRL/*lpr* mice. The results shown are representative of at least four independent experiments.

CD39/ENTPD1, an ectoenzyme that degrades extracellular ATP to AMP, is highly expressed on the majority of monocytes and B cells and a fraction of CD4^+^ T cells, but not on CD8^+^ T cells [37]. To examine whether the loss of P2X7R response of the B220^+^ T cells is due to enhanced hydrolysis of ATP following CD39 overexpression, we have analysed the levels of CD39 membrane expression in CD19^+^ B cells, B220^–^CD90^+^ T cells (either CD4^+^ or CD8^+^) and B220^+^CD90^+^ DN T cells from MRL/*lpr* mice. Flow cytometry analyses show that CD39 levels are significantly higher on B220^–^CD4^+^ T cells than in B220^–^CD8^+^ T cells and B220^+^ DN T cells (Fig. 8A). Since B220^−^CD4^+^ T cells from MRL/*lpr* mice respond to exogenously added ATP though expressing high levels of CD39 at the plasma membrane, it is very unlikely that a lower level of CD39 expression on B220^+^ DN T cells could account for the lack of activation of P2X7R in B220^+^ T lymphocytes.

### P2X7R is not Expressed on the Cell Surface of B220^+^ T Cells

The level of P2X7R membrane expression is a limiting factor for signaling, and correlate with the threshold of activation of P2X7R [38]. Therefore, we quantified P2X7R at mRNA and protein levels by real-time RT-PCR, western blot and flow cytometry in LN cells or FACS-purified B220^–^ and B220^+^ CD90^+^ T lymphocytes from B6, MRL^+/+^, MRL/*lpr* and B6*^P2X7R−/−^* mice. RT-PCR and western blot analyses show that B220^+^ T cells from MRL/*lpr* mice express high levels of P2X7R mRNA (Fig. 8B) and proteins (Fig. 8C) that were similar or even higher than in B220^–^ T cells. As expected, no P2X7R proteins were detected in B6*^P2X7R−/−^* mice (Fig. 8C). The absence of P2X7R activity in MRL/*lpr* T cell population did not seem to correlate with a lower expression of P2X7R protein. Therefore, we examined whether the absence of P2X7R response in B220^+^ T cells could be due to reduced expression of P2X7R at the plasma membrane. Using rabbit polyclonal antiserum that recognizes conformational epitope on P2X7R and flow cytometry, we determined the expression of P2X7R on B220^−^ T cells (either CD4^+^ or CD8^+^) from MRL*^+/+^* (Fig. 8D) and MRL/*lpr* mice as well as on B220-expressing CD4^+^, CD8^+^ and DN T-cell subsets from MRL/*lpr* mice (Fig. 8E). The staining patterns obtained with CD4^+^ and CD8^+^ T cells from B6*^P2X7R−/−^* mice were used to set background fluorescence for P2X7R expression, as shown in MRL^+/+^ plots (Fig. 8D). B220^−^ T cells (either CD4^+^ or CD8^+^) from MRL^+/+^ and MRL/*lpr* mice expressed P2X7R, albeit at different levels (based on MFI values) (Fig. 8D and 8E). In contrast, no P2X7R labeling was observed on B220^+^ DN T cells as well as on B220 positive CD4^+^ and CD8^+^ T cells from MRL/*lpr* mice (Fig. 8E), indicating that the defect in P2X7R signaling in B220^+^ T cells is due to a lack of P2X7R at the cell surface.

## Discussion

Our present study shows that 1) the B220^+^ DN T cells that accumulate in spleen and LNs of autoimmune MRL/*lpr* mice due to the *fas* mutation have an impaired P2X7R pathway, 2) the few B220^+^CD4^+^ and B220^+^CD8^+^ T cells found in the spleen and LNs of normal B6 and MRL^+/+^ mice have also an impaired P2X7R pathway, 3) while similar levels of P2X7R proteins are detected by western blot in LN cells from MRL^+/+^ and MRL/*lpr* mice, flow cytometric analysis revealed that P2X7R were not actually expressed on the plasma membrane of B220^+^ T cells (either CD4^+^, CD8^+^ or DN). Our present report supports the following important points: first, the loss of P2X7R activity in B220^+^ DN T cells from MRL/*lpr* mice could amplify the defect in peripheral T cell homeostasis due to the *fas* mutation and thus contribute to the autoimmune pathology. However, the role of P2X7R in SLE is difficult to evaluate since several other loci are involved in the development and severity of the disease [15], [16]. Second, the lower activity of P2X7R in normal B220^+^ T cells could be a physiological regulatory mechanism.

During infections, protective immunity produces inflammatory cytokines and killer cells to destroy invading microorganisms, but also causes the destruction of normal cells. Infected or stressed cells release ATP into the extracellular compartment, which activates P2X7R. P2X7R activation on immune cells causes inflammation, including cytokine secretion and dendritic cell activation [39]. P2X7R activation also induces shedding of LN homing receptor CD62L from the cell surface of leukocytes [27], [40], regulating leukocyte extravasation in inflamed tissues. Furthermore, CD4^+^CD25^+^ regulatory T cells (Treg), which are resistant to apoptosis via their antigen receptor, die *in vivo* in the presence of extracellular ATP [41], [42] or NAD [43], enabling a more efficient immune response in inflammatory sites. However, human and murine CD4^+^ Treg could be protected, to some extent, from cell death induced by extracellular ATP or NAD because they constitutively express ATP/ADPase CD39, AMPase CD73, and NADase CD38 on the plasma membrane [37], [43], [44]. Indeed, Tregs play a critical role in maintaining immune homeostasis and preventing autoimmune diseases [45], and their elimination by extracellular ATP and NAD might be responsible for breaking tolerance in inflamed tissues. Other subsets of regulatory T cells have been identified: CD8^+^ T cells, natural killer T (NKT) cells and DN T cells [3], [46]. DN Tregs are generated in the periphery during a primary immune response, and kill effector T cells in an antigen-specific manner through Fas/FasL interactions [3], [46]. Interestingly, it has been suggested that part of the B220^+^ DN T cells found in autoimmune MRL/*lpr* mice represents Treg cells [47], [48].

Autoimmune disease also arises from defective homeostatic regulation of effector T cells [1]. It is largely recognized, that human and murine effector T cells upregulate B220 molecules before undergoing apoptosis by the Fas pathway [5], [6], [49]. In MRL/*lpr* mice or ALPS patients, effector T cells overexpress B220 at their surface though the Fas pathway is defective. The significant association between the overexpression of B220 and loss of P2X7R expression we identified on T cells from both wild-type and Fas-deficient mice lead us to suggest that purinergic P2X7R is involved in a T-cell homeostatic pathway different of that of death receptor Fas. The inactivation of the P2X7R pathway in B220^+^ DN T cells could amplify in MRL/*lpr* mice the lymphadenopathy due to impaired Fas-mediated apoptosis. Indeed, when effector T cells acquire B220 surface expression, they are protected from death triggered by extracellular ATP or NAD released by inflamed tissues and unable to cleave CD62L, although ADAM17 protease responsible for CD62L shedding [28] is functional in these T cells (Fig. 4). Therefore, they are able to return to LNs where they accumulate.

Interestingly, another isoform of the transmembrane tyrosine phosphatase CD45 has been found to be associated with the regulation of P2X7R functions. The sensitivity of CD4^+^ T cells to ATP-induced PS externalization and cell death appears to correlate with the levels of CD45RB membrane expression. CD4^+^ T cells expressing low levels of CD45RB are more responsive to ATP stimulation than their counterpart expressing high levels of CD45RB [42], [50]. High and low levels of CD45RB expression on mouse T cells are a feature of naïve cells and antigen-activated cells, respectively [7], [51], suggesting that activated T cells (CD45RB*^low^*) are more sensitive to ATP stimulation than naïve T cells (CD45RB*^high^*). In B220^+^ (or CD45RABC*^high^*) DN T cells, we exclude a role for CD45RB in the loss of P2X7R functions since B220^+^ DN T display a central memory (CD45RB*^low^*CD62L*^high^*CD44*^high^*) phenotype [7], [52].

In summary, mutations in a single gene, namely *fas*, promote a T-cell lymphoproliferation and the constitutive expression of B220 on T cells, which correlates with a marked downregulation of cell surface P2X7R expression and, therefore, an impaired functionality of P2X7R. The high numbers of abnormal B220^+^ DN T cells in MRL/*lpr* mice totally mask the response of normal B220^−^ T-cell subsets (CD4^+^ or CD8^+^) to P2X7R stimulation. Elimination of these pathogenic B220^+^ DN T cells *in vivo* by As_2_O_3_ treatment restored the response of MRL/*lpr* T-cell population to P2X7R stimulation and normal T-cell homeostasis. Likewise, a previous report had shown that the inactivation of B220 in FasL-deficient mice improved the lymphoproliferative disorder [53]. A number of susceptibility loci for SLE have been mapped, but the key question is how each genetic factor contributes to the disease. We hypothesize that the defect in the homeostasis of activated *lpr* T cells, due to the inactivation of the Fas/FasL pathway, is amplified by the impaired activity of P2X7R in B220^+^ T cells.

## Supporting Information

Figure S1 **NAD-induced shedding of CD62L and PS externalization in T lymphocytes from MRL^+/+^ and MRL/lpr mice.** Spleen cells from MRL*^+/+^* and MRL/*lpr* mice (n = 3 mice/group) were either left unstimulated (□) or stimulated with 50 µM NAD (▪) for 45 min at 37°C. Cells were then double-stained with anti-CD90 mAb and either anti-CD62L mAb or Annexin V to assess CD62L shedding and PS exposure, respectively, in the gated CD90^+^ T-cell population by flow cytometry.
(TIF)

Figure S2 **P2X7R activity in T lymphocytes from normal MRL^+/+^ and autoimmune MRL/lpr mice treated by arsenic trioxide for two months.** Two-month-old MRL*^+/+^* (□) and MRL/*lpr* (▪) mice (n = 3 mice/group) have been treated five days a week for 2 months with PBS or As_2_O_3_ (5 µg/g). Spleen cells from treated mice were then incubated with or without 500 µM ATP for 45 min at 37°C. Spleen cells were then triple-stained with anti-CD90 and anti-CD19 mAb and either anti-CD62L mAb or YO-PRO-1 to assess CD62L shedding (A) and pore formation (B), respectively, in CD19^–^CD90^+^ T cells by flow cytometry. Asterisks denote statistically significant differences between ATP-stimulated and unstimulated groups: ***p≤0.001.
(TIF)

Figure S3 **P2X7R antagonist KN-62 inhibits ATP-induced CD62L shedding and pore formation in B220^–^ T cells from MRL/lpr mice.** Spleen cells from 3-mo-old MRL*/lpr* mice preincubated at 37°C for 15 min with or without 0.5, 1 and 5 µM P2X7R inhibitor KN-62 were stimulated or not by 500 µM ATP for 45 min at 37°C. Cells were then triple-stained with anti-CD90, anti-B220 and either anti-CD62L mAb or YO-PRO-1 to assess CD62L shedding (A) or pore formation (B) in B220^–^ (□) and B220^+^ (▪) CD90^+^ T cells. Results are representative of three independent experiments.
(TIF)
